# Metabolomic Analysis Identifies Glycometabolism Pathways as Potential Targets of Qianggan Extract in Hyperglycemia Rats

**DOI:** 10.3389/fphar.2020.00671

**Published:** 2020-05-12

**Authors:** Mingzhe Zhu, Meng Li, Wenjun Zhou, Guangbo Ge, Li Zhang, Guang Ji

**Affiliations:** ^1^ Institute of Digestive Diseases, China-Canada Center of Research for Digestive Diseases (ccCRDD), Longhua Hospital, Shanghai University of Traditional Chinese Medicine, Shanghai, China; ^2^ School of Public Health, Shanghai University of Traditional Chinese Medicine, Shanghai, China; ^3^ Institute of Interdisciplinary Integrative Medicine Research, Shanghai University of Traditional Chinese Medicine, Shanghai, China

**Keywords:** Qianggan extract, hyperglycemia, glycometabolism, metabolomics, gas chromatography-mass spectrometry

## Abstract

Qianggan formula, a designed prescription according to the Traditional Chinese Medicine (TCM) theory, is widely used in treating chronic liver diseases, and indicated to prevent blood glucose increase in patients *via* unknown mechanisms. To unravel the effects and underlying mechanisms of Qianggan formula on hyperglycemia, we administrated Qianggan extract to high fat and high sucrose (HFHS) diet rats. Results showed that four-week Qianggan extract intervention significantly decreased serum fasting blood glucose, hemoglobin A1c, and liver glycogen levels. Gas chromatography-mass spectrometry (GC-MS) approach was employed to explore metabolomic profiles in liver and fecal samples. By multivariate and univariate statistical analysis (variable importance of projection value > 1 and *p* value < 0.05), 44 metabolites (18 in liver and 30 in feces) were identified as significantly different. Hierarchical cluster analysis revealed that most differential metabolites had opposite patterns between pair-wise groups. Qianggan extract restored the diet induced metabolite perturbations. Metabolite sets enrichment and pathway enrichment analysis revealed that the affected metabolites were mainly enriched in glycometabolism pathways such as glycolysis/gluconeogenesis, pentose phosphate pathway, fructose, and mannose metabolism. By compound-reaction-enzyme-gene network analysis, batches of genes (e.g*. Hk1, Gck, Rpia*, *etc*) or enzymes (e.g. hexokinase and glucokinase) related to metabolites in enriched pathways were obtained. Our findings demonstrated that Qianggan extract alleviated hyperglycemia, and the effects might be partially due to the regulation of glycometabolism related pathways.

## Introduction

Traditional Chinese medicine (TCM) has been used in clinical applications for thousands of years (Zhang et al., 2016b). TCM formulae are mainly composed of herbs and widely used to treat metabolic diseases, such as hepatic steatosis and type 2 diabetes (Liang et al., 2018; Yu et al., 2018). Herbal extracts from traditional Chinese medicines such as curcumin, capsaicin and ginsenosides have been effectively employed in preventing obesity and other metabolic diseases (Yu et al., 2018). Qianggan formula is a patent TCM drug, and composed of 16 ingredients. Qianggan formula has been implicated in clinical practice and proved to be effective in improving metabolic disease (Li et al., 2010; Gu and Huang, 2011; Wang et al., 2011). However, little has been reported the mechanisms underlying the efficacy, which needs to be clarified.

TCM is a holistic system, which comprises multicomponent complexes and has multiple therapeutic targets (Li et al., 2017). It would be helpful to apply systemic approaches to elucidate the underlying mechanisms. Metabolomics is an important part of systems biology and provides global information of small molecule metabolites in complex biological processes (Crowther et al., 2018). It offers a powerful platform to investigate metabolic pathways, identify biomarkers for diagnosing and monitoring diseases, and predict therapeutic targets of drugs (Guo et al., 2018; Procopet et al., 2018). Gas chromatography-mass spectrometry (GC-MS), which possesses high resolution, sensitivity, and available database, is one of the powerful and popular tools in metabolomics studies (Shackleton et al., 2018). It has been extensively applied to assess the effects and explore metabolic mechanisms of TCM in treating diseases. By GC-MS based plasma metabolomics, Feng D, et al. identified potential biomarkers and established metabolic networks to explain the efficacy of Xuefu Zhuyu Decoction on traumatic brain injury (Feng et al., 2017). Gou XJ, et al. employed GC-MS to elucidate the underlying mechanisms of Qushi Huayu Decoction in a fatty liver rat model and obtained 23 potential biomarkers and several regulating metabolic pathways (Gou et al., 2017). Another study using GC-MS implicated the important roles of three carbohydrate metabolism pathways of Hedyotis diffusa decoction in preventing acute liver injury (Dai et al., 2017).

In the present study, GC-MS based metabolomics (liver and fecal samples) was employed to evaluate metabolic alterations of high fat and high sucrose (HFHS) diet fed rats, and obtain Qianggan extract affected metabolites. With the aid of pattern recognition, metabolite set enrichment analysis (MSEA), pathway enrichment analysis and compound-reaction-enzyme-gene network analysis, potential candidate metabolites, and relevant metabolic pathways were identified. Our study inferred the mechanisms of Qianggan extract on hyperglycermia and suggested a new pattern for studying TCM formula on metabolic diseases.

## Material and Methods

### Preparation of Qianggan Extract

Qianggan formula is a marketed TCM, which was prepared by 16 herbal materials. In this study, the Qianggan extract was prepared as previously reported (Zhu et al., 2019). Briefly, all of the ingredients: *Artemisia scoparia* Waldst. & Kitam. (Yin-Chen) 250 g*, Isatis tinctoria* L. (Ban-Lan-Gen) 125 g, *Angelica sinensis*(Oliv.)Diels. (Dang-Gui) 125 g, *Paeonia lactiflora* Pall. (Bai-Shao) 125 g, *Salvia miltiorrhiza* Bunge. (Dan-Shen) 250 g, *Curcuma wenyujin* Y.H.Chen et C.Ling. (Yu-Jin) 125 g, *Astragalus membranaceus*(Fisch.)Bunge. (Huang-Qi) 250 g, *Codonopsis pilosula*(Franch.)Nannf. (Dang-Shen) 125 g, *Alisma orientale*(Sam.)Juz. (Ze-Xie) 125 g, *Polygonatum kingianum* Collett& Hemsl. (Huang-Jing) 125 g, *Rehmannia glutinosa* (Gaertn.) DC. (Shen-Di) 125 g, *Dioscorea oppositifolia* L. (Shan-Yao) 125 g, *Crataegus pinnatifida* Bunge.(Shan-Zha) 100 g, *Medicated Leaven Massa Medicata Fermentata* (Liu-Shen-Qu) 100 g, *Gentiana macrophylla* Pall.(Qin-Jiao) 100 g, *Glycyrrhiza uralensis* Fisch. (Gan-Cao) 100 g were mixed and soaked in water, and then boiled for 2 h. These herbal materials were extracted by hot-water for three times, then mixed and filtrated to get the supernatants. After then, the pH of the supernatants was adjusted to 8.0, and concentrated the solution to a density ratio of 1.35 to obtain the Qianggan water extract. The extract was re-dissolved in acetonitrile-water (1:1, v/v) for chemical profiling analysis. A Agilent 1290 UPLC system (Agilent Technologies, Palo Alto, USA) coupled with Sciex TripleTOF 4600^®^ quadrupole-time of flight mass spectrometer (AB Sciex, Darmstadt, Germany) equipped with a DuoSpray source was used for profiling the chemical constituents in Qianggan extract. Chromatographic separation was achieved on an Acquity UPLC^®^ HSS T3 column (2.1×100 mm, 1.7 μm; Waters, Milford, MA, USA). The mobile phase consisted of water containing 0.1% formic acid (A) and acetonitrile (B). The following gradient condition was used: 0–3.0 min, 0% B; 3.0–5.0 min, 0% B-5% B; 5.0–7.0 min 5% B-15% B; 7.0–21.0 min, 15% B-30% B; 21.0-24.0 min, 30% B–48% B; 24.0-30.0 min, 48% B–60% B; 30.0-34.0 min, 60% B-95% B; 34.0–36.0 min, 95% B; 36.0–36.1 min, 95% B-0% B; 36.1–40.0 min, 0% B. The injection volumes for all samples were 5 μl. Column oven temperatures was set at 30 °C, while the flow rate was 0.3 ml/min. Ionization was conducted using an electrospray ionization (ESI) source. Data were collected under both positive and negative ion modes. The mass spectrometer was operated in full-scan TOF-MS at m/z 100-1500 and information-dependent acquisition (IDA) MS/MS modes, the collision energy was 40 ± 20 eV. Both ion source gas 1 and 2 were set 50 psi. Curtain gas was 35 psi. The temperature and ionspray voltage floating were 500°C and 5000/-4500 V, respectively. Data recording and processing was performed by Analyst Ver. 1.6 software (AB Sciex, USA).

### Animal Experiments and Sample Collection

Six-week-old male Wistar rats were purchased from Shanghai SLAC Laboratory Animal Co. Ltd, China, and maintained in specific pathogen free (SPF) environment. According to the body weight, 24 rats were randomly divided into normal group (n=8), fed with chow diet, and HFHS group (n=16), fed with a diet composing 68% chow diet, 15% lard, 15% sucrose, and 2% cholesterol; After 6-week feeding, HFHS rats were further divided into untreated group (HFHS, n=8), and Qianggan extract intervened group (n=8) that fed with HFHS diet and administered with Qianggan extract that dissolved in distilled water (1.2 g/kg/d) *via* gavage. The rats were allocated with 4 per cage, and fed and/or intervened for another 4 weeks. At the end, animals were weighed after 12 h-fasting, euthanized with 2% pentobarbital sodium, and sacrificed. Blood was collected and serum was separated. The livers were weighed, divided into portions, and stored at -80°C.

The study was carried out in accordance with the recommendations of National Institutes of Health Guidelines for the Care and Use of Laboratory Animals. The protocol was approved by the Animal Ethics Committee of Shanghai University of Traditional Chinese Medicine (PZSHUTCM191227006).

### Serum Biochemical Analysis

Serum alanine transaminase (ALT), aspartate transaminase (AST), triglyceride (TG), total cholesterol (TC), low density lipoprotein cholesterol (LDL-c), and blood glucose (BG) were analyzed using the Hitachi full-automatic system. Serum insulin and hemoglobin A1c (HbA1c) were analyzed by enzyme-linked immunosorbent assay (ELISA). Serum insulin and BG were used to calculate the homeostasis model assessment of insulin resistance (HOMA-IR).

### Detection of Liver Glycogen

Liver glycogen was measured by commercial kit (Jiancheng Tech, Nanjing, China) according to the instructions of the manufacturer. Briefly, 25 mg of liver tissue were pretreated with 30% KOH, ethanol, and saturated sodium sulfate. After collecting the supernatants, reagent anthrone and neutralized hydrolysate were added. The final solutions of reaction were read in microplate reader at 620 nm.

### GC-MS Based Metabolomics Analysis

Sample preparation, GC-MS metabolomics analysis, and metabolite identification of liver tissue and feces were conducted by Shanghai Profleader Biotech Co., Ltd (Shanghai, China). After adding 20-fold volume (μl/mg) of chloroform/methanol/water solvent (v/v/v=2:5:2) containing 10 μg/ml of L-norvaline and freezing at -40°C for 30 min, the frozen liver tissue samples were ground immediately by using a TissueLyser (type JX-24, Jingxin, Shanghai, China) with zirconia beads for 3 min at 50 Hz. The homogenates were incubated at -20°C for an hour, followed by vortex and centrifugation at 14,000 g and 4°C for 15 min. The extraction was repeated with methanol as solvent and the supernatants from the two extractions were combined. The combined supernatants (100 μl) and ^13^C_6_-^15^N-L-isoleucine (10 μl) were blended and dried under nitrogen gas. For the extraction of feces sample, a frozen feces sample was strongly vortexed in 10-fold volume (μl/mg) of ice-cold deionized water containing 10 μg/ml of ^13^C_4_-succinic acid, and then incubated at 4°C for 30 min. Following centrifugation at 16,000 g and 4°C for 15 min, the supernatant was collected. The extraction was repeated with deionized water as solvent and the supernatants from the two extractions were combined, followed by protein precipitation with four-fold volume (v/v) of methanol. After centrifugation, 500 μl combined supernatants were mixed and evaporated to dryness under nitrogen stream. The dried residues of liver or feces were dissolved in 30 μl methoxyamine hydrochloride in pyridine (20 mg/ml) and then incubated at 37°C for 90 min. After an addition of 30 μl BSTFA (with 1% TMCS), the sample was derivatized at 70°C for 60 min prior to GC-MS analysis. Quality control (QC) sample pooled from all samples were prepared and analyzed with the same procedure as those of the experiment samples. Blank samples were also prepared where sample was replaced by deionized water so as to monitor and remove the contaminants introduced during sample preparation and column bleeds.

Metabolomics analysis was conducted on an Agilent 7890A gas chromatography system coupled to an Agilent 5975C inert MSD system (Agilent Technologies Inc., CA, USA). The experiments were performed following the previously described protocol (Liu et al., 2018). Raw data were obtained in a full scan mode. The samples were run at random, and blank samples and QC samples were inserted during sample analysis.

### Data Processing, Pattern Recognition, and Metabolites Structure Identification

GC-MS raw data were processed by TagFinder software (Luedemann et al., 2008) according to previously published methods (Gao et al., 2010). The final data was obtained, which included sample names, variables (rt_mz), and peak abundances. The added internal standards were utilized to monitor the GC-MS signal fluctuation during sample analysis. The metabolite peaks with relative standard deviation (RSD) value of abundances in QC samples larger than 30% were filtered out. After filtering, the qualified data were performed median normalization before performing further univariate and multivariate statistics.

Fold change was calculated as binary logarithm of average normalized peak intensity ratio between groups. To better understand the pattern of differential metabolites among groups, hierarchical clusters were performed by Cluster 3.0 software. Venn diagram of identified metabolites between liver and feces samples was visualized by a web tool (bioinformatics.psb.ugent.be/webtools/Venn/).

To identify the structure of differential metabolites, GC-MS raw data were imported to AMDIS software and the purified mass spectra were compared to an in-house standard library, Golm Metabolome Database, and Agilent Fiehn GC-MS Metabolomics RTL Library.

### Metabolite Set Enrichment Analysis (MSEA) and Pathway Analysis

To identify biologically meaningful patterns and most relevant metabolic pathways of the differential metabolites, MSEA and pathway enrichment analysis were performed by MetaboAnalyst 4.0 (http://www.metaboanalyst.ca/) as previously described (Chong et al., 2018). To demonstrate the relationships among genes, proteins, and metabolites in related pathways, Compound-Reaction-Enzyme-Gene network was constructed by Cytoscape software plug-in Metscape (Karnovsky et al., 2012).

### Statistical Analysis

By SIMCA software (version 14.1, Umetrics, Umeå, Sweden), principle component analysis (PCA) and orthogonal partial least squares discriminant analysis (OPLS-DA) were conducted for multivariate statistical analysis, where the data were preprocessed by unit variance (UV) scaling and mean centering. The model quality is estimated by R2X or R2Y and Q2 values. To avoid OPLS-DA model over-fitting, 200 times permutation tests were carried out. Then variable importance of projection (VIP) values were visualized in OPLS-DA model. For univariate statistical analysis, Welch's t test was conducted on the data of normal distribution, while Wilcoxon Mann-Whitney test was conducted on the data of abnormal distribution. Finally, the metabolites with VIP > 1 and p < 0.05 were identified as different metabolites.

Data were expressed as mean ± SD and were analyzed by one-way analysis of variance (ANOVA) by SPSS v22.0 software. P value less than 0.05 was considered as statistically different.

## Results

### Chemical Profiling of Qianggan Extract

**Figure 1:**
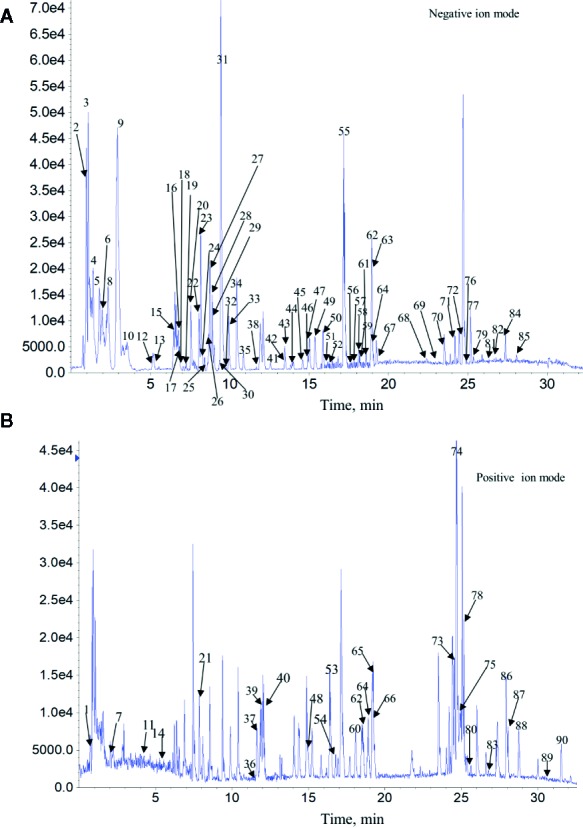
Total ion chromatogram of constitutes in Qianggan extract. Agilent 1290 UPLC system was applied to analyze the chemical profiling of Qianggan extract, data were collected under both negative ion mode **(A)** and positive ion mode **(B)**, and processed by Analyst Ver. 1.6 software.

The complexity of MS data acquired in both mass spectrometry (MS) and tandem mass spectrometry (MS/MS) mode requires reliable peak identification tools. In this work, SCIEX natural products HR-MS/MS Spectral Library was used for identification. The library contains additional compound entries with structural information and MS/MS spectra for 1,300 selected natural compounds. The assignment of each constitute was performed by comparing the retention times (Rt), MS data (accurate mass, isotopic distribution, and fragmentation pattern) of each constitute from Qianggan extract with SCIEX natural products HR-MS/MS Spectral Library (involving compound name, molecular formula, chemical structure, CAS No, accurate MS/MS spectra, *ect*.) and previously reported literature (Cao et al., 2011). With the help of PeakView 1.2 and MasterView 1.1, a total of 90 constitutes have been identified or tentatively characterized in Qianggan extract (compounds of 16 herbs) under positive or negative ion mode (**Figure 1** and **Table 1**). Among them, 4 constitutes attributed to *Cynanchum otophyllum,* 6 attributed to *Radix isatidis,* 6 attributed to *Radix Angelicae sinensis,* 10 attributed to *Radix Paeoniae Alba,* 17 attributed to *Radix Salviae miltiorrhizae,* 2 attributed to *Curcuma aromatic,* 10 attributed to *Astragalus membranaceus,* 2 attributed to *Codonopsis pilosula,* 2 attributed to *Rhizoma alismatis,* 2 attributed to *Rehmannia glutinosa,* 7 attributed to *Rhizoma Dioscoreae,* 3 attributed to *Hawthorn,* 7 attributed to *Medicated Leaven,* 10 attributed to *Fraxinus bungeana,* 23 attributed to *Radix liquiritiae.* Un-expectably, no metabolite was detected from *Rhizoma polygonat* (**Table 1**).

**Table 1:** The detected ion chromatogram of constitutes in Qianggan extract.

No.	Retention time (min)	Name	Formula	Ion	Measured m/z	Calculated m/z	Error (ppm)	Product ion (m/z)	Attribution
1	0.84	Arginine	C6H14N4O2	[M+H]^+^	175.1194	175.1190	2.6	175.1197; 116.0707; 70.0646; 60.0558	Radix isatidis, Rhizoma Dioscoreae
2	1.01	Gentianose	C18H32O16	[M+FA-H]^-^	549.1685	549.1672	2.3	549.1627; 503.1618; 341.1129; 221.0645; 179.0583; 143.0365; 89.0232; 59.0125	Fraxinus bungeana
3	1.09	Stachyose tetrahydrate	C24H42O21	[M-H]^-^	665.2150	665.2146	0.6	665.2138; 485.1512; 443.1401; 383.1181; 341.1066; 221.0651; 179.0551; 161.0443; 101.0234	Radix Salviae miltiorrhizae
4	1.39	Sucrose	C12H22O11	[M-H]^-^	341.1072	341.1089	-5.1	341.1068; 179.0541; 149.0442; 115.0031; 89.0228; 71.0129	Radix Paeoniae Alba, Radix Paeoniae Alba
5	1.76	inulin	C24H42O21	[M+FA-H]^-^	711.2177	711.2201	-2.2	711.2218; 665.2181; 485.1483; 383.1194; 341.1081; 179.0551; 89.0233	Codonopsis pilosula
6	1.95	Maltotriose	C18H32O16	[M-H]^-^	503.1605	503.1618	-2.5	503.1569; 341.1056; 281.0862; 221.0631; 179.0540; 89.0234	Radix Salviae miltiorrhizae
7	1.99	uracil	C4H4N2O2	[M+H]^+^	113.0344	113.0346	-1.4	/	Radix Paeoniae Alba
8	2.34	Raffinose	C18H32O16	[M-H]^-^	503.1604	503.1618	-2.7	503.1556; 341.1020; 221.0641; 179.0526; 143.0338; 119.0318; 89.0228; 59.0132	Codonopsis pilosula, Radix Salviae miltiorrhizae
9	2.94	Citric acid	C6H8O7	[M-H]^-^	191.0190	191.0197	-3.8	191.0184; 111.0070; 87.0065	Hawthorn, Radix Paeoniae Alba
10	3.54	2-O-a-D-glucopyranuronosyl-D-Galactose	C12H20O12	[M-H]^-^	355.0863	355.0882	-5.4	355.0873; 181.0726; 173.0070; 111.0073; 87.0072; 57.0359	Radix Salviae miltiorrhizae
11	3.82	tyrosine	C9H11NO3	[M+H]^+^	182.0817	182.0812	2.9	/	Rhizoma Dioscoreae
12	5.16	Uridine	C9H12N2O6	[M-H]^-^	243.0611	243.0623	-4.8	243.0625; 200.0559; 152.0357; 110.0234; 82.0315	Radix isatidis
13	5.17	verbascotetraose	C24H42O21	[M+FA-H]^-^	711.2186	711.2201	-2.1	711.2238; 665.2139; 485.1462; 323.0960; 179.0536; 143.0337	Rhizoma alismatis
14	6.39	Adenosine	C10H13N5O4	[M+H]^+^	268.1043	268.1040	1.0	268.1026; 136.0617; 119.0363	Radix Angelicae sinensis, Radix Paeoniae AlbaRadix Paeoniae Alba, Medicated Leaven
15	6.53	verbascose or isomer	C30H52O26	[M-H]^-^	827.2659	827.2674	-1.8	827.2655; 665.2270; 647.1999; 503.1571; 485.1452; 383.1153; 341.1031; 179.0544; 161.0431; 143.0331	Rehmannia glutinosa, Rhizoma alismatis
16	6.63	Dicaffeoyl quinic acid glucoside	C31H34O17	[M-H]^-^	677.1776	677.1723	6.5	677.1765; 479.1077; 341.1034; 173.0074; 111.0064;	Cynanchum otophyllum
17	6.84	Guanosine	C10H13N5O5	[M-H]^-^	282.0827	282.0837	-6.0	282.0837; 150.0415; 133.0151; 108.0182	Radix isatidis, Medicated Leaven
18	6.99	Rehmannioside D	C27H42O20	[M+Cl]^-^	721.1911	721.1963	-7.3	721.1913; 263.0765; 221.0662; 179.0565; 149.0448; 119.0346; 89.0216	Rehmannia glutinosa
19	7.48	L-Alanine	C15H24N4O5	[M+H]^+^	341.1814	341.1819	-1.6	341.1809; 281.1604; 222.1125; 194.1171; 108.0798; 87.0443	Radix Angelicae sinensis, Rhizoma Dioscoreae
20	7.54	6'-O-acetylscopolin	C18H20O10	[M-H]^-^	385.0966	395.0984	-4.5	197.0440; 179.0334; 135.0443; 123.0437; 72.9932	Hawthorn
21	7.86	(1,2,4-Triazolo[4,3-a]pyrazine-3,7(8H)-dicarboxylic acid, 5,6-dihydro-, 7-(1,1-dimethylethyl) 3-ethyl ester)	C13H20N4O4	[M+H]+	297.1560	297.1557	0.9	297.1514; 279.1415; 219.1114; 192.1014; 232.0799; 117.0562; 108.0448; 70.0650	Radix Angelicae sinensis
22	8.07	NeoChlorogenic acid	C16H18O9	[M-H]^-^	353.0885	353.0878	2.0	353.0904; 191.0553; 179.0336; 135.0447; 85.0289	Radix Angelicae sinensis
23	8.18	Loganic acid or isomer	C16H24O10	[M-H]^-^	375.1298	375.1297	0.3	375.1325; 213.0779; 169.0874; 113.0248; 59.0137	Fraxinus bungeana
				[M+Na]+	399.1262	399.1262	0.1	399.1256; 381.1205; 279.0706; 237.0698; 219.0615; 185.0417; 112.0864	
24	8.21	Desbenzoylpaeoniflorin	C16H24O10	[M-H]^-^	375.1289	375.1297	-2.1	375.1290; 213.0773; 169.0885; 151.0766; 113.0254; 89.0247; 69.0344	Radix Paeoniae Alba
25	8.65	salicylicacid	C7H6O3	[M-H]^-^	137.0238	137.0244	-4.5	137.0244; 92.0274	Radix isatidis
26	8.67	ArillatoseB	C22H30O14	[M+FA-H]^-^	563.1626	563.1618	1.5	563.1598; 517.1596; 341.1119; 251.1756; 221.0637; 179.0552; 161.0438; 119.0344; 89.0243	Fraxinus bungeana
				[M+Na]^+^	541.1526	541.1528	-0.3	541.1537; 497.1683; 393.1005; 365.1040; 347.0938	
27	8.74	Chlorogenic acid	C16H18O9	[M-H]^-^	353.0880	353.0878	0.5	191.0563; 85.0292	Radix Angelicae sinensis
28	8.84	Swertiamain	C16H22O10	[M+FA-H]^-^	419.1196	419.1195	0.2	375.0668; 179.0551; 141.0186; 119.0382; 89.0253	Fraxinus bungeana
29	8.90	Chlorogenic acid isomer	C16H18O9	[M-H]^-^	353.0881	353.0878	0.6	353.0908; 191.0573; 173.0466; 135.0460; 93.0343; 85.0300	Radix Angelicae sinensis
30	9.42	Kaempferol 3-rutinoside	C27H30O15	[M-H]^-^	593.1498	593.1512	-2.4	593.1508; 575.1468; 503.1116; 473.1058; 383.0763; 353.0642	Radix isatidis, Medicated Leaven
31	9.44	Gentiopicroside	C16H20O9	[M+FA-H]^-^	401.1098	401.1089	2.2	401.1064; 193.0480; 179.0577; 149.0597; 113.0239; 89.0234; 59.0122	Fraxinus bungeana
				[M+Na]+	379.0999	379.1000	-0.1	379.1004; 217.0469; 199.0358; 185.0442; 155.0456	
32	9.73	Artemisinin	C15H22O5	[M-H]^-^	281.1377	281.1394	-6.2	/	Medicated Leaven
33	9.94	Albiflorin	C23H28O11	[M+FA-H]^-^	525.1629	525.1614	2.9	525.1586; 479.1544; 283.0819; 121.0290; 77.0390	Radix Paeoniae Alba
				[M+Na]+	503.1523	503.1524	-0.2	503.1534; 341.0989	
34	10.45	Paeoniflorin	C23H28O11	[M+FA-H]^-^	525.163	525.1614	3.1	525.1695; 449.1462; 431.1358; 327.1094; 309.0994; 165.0553; 121.0295; 113.0237; 77.0402	Radix Paeoniae Alba
				[M+Na]+	503.1527	503.1524	0.6	503.1531; 341.1050	
35	10.86	5-Hydroxy ferulic acid	C10H10O5	[M-H]^-^	209.0447	209.0455	-4.1	209.0458; 165.0536; 121.0273; 76.0302	Fraxinus bungeana
36	11.28	Agarotetrol	C17H18O6	[M+H]+	319.1174	319.1176	-0.7	319.1163; 301.1055; 283.0968; 255.1025; 227.1084; 192.0403; 164.0483; 125.0259; 91.0548	Medicated Leaven
37	11.65	Calycosin-7-O-D-glucoside	C22H22O10	[M+H]+	447.1285	447.1286	-0.2	447.1251; 343.0117; 285.0749; 270.0521; 225.0553	Curcuma aromatic
38	11.81	Rutin	C27H30O16	[M-H]^-^	609.1468	609.1461	1.1	/	Medicated Leaven
39	11.93	Isoliquiritin apioside	C26H30O13	[M-H]^-^	549.1624	549.1614	1.9	549.1608; 429.1245; 255.0668; 135.0098; 119.0500	Radix liquiritiae
				[M+Na]+	573.158	573.1579	0.2	573.1611; 441.1097; 317.0843	
40	12.1	Liquiritin	C21H22O9	[M-H]^-^	417.1196	417.1191	1.2	417.1188; 255.0652; 135.0091; 119.0506	Radix liquiritiae
				[M+Na]+	441.1158	441.1156	0.4	441.1152	
41	12.55	Galloylpaeoniflorin	C30H32O15	[M-H]^-^	631.1675	631.1668	1.0	631.1697; 613.1596; 465.1354; 399.0920; 313.0558; 271.0492; 211.0313; 169.0139	Radix Paeoniae Alba
42	13.47	1,5-Dicaffeoyl quinic acid	C25H24O12	[M-H]^-^	515.1204	515.1195	1.7	515.1171; 353.0843; 335.0722; 191.0566; 179.0353; 173.0573; 161.0240; 135.0451	Cynanchum otophyllum
43	13.59	Verbascoside	C29H36O15	[M-H]^-^	623.1963	623.1981	-3.0	623.1894; 461.1569; 161.0238; 133.0329	Curcuma aromatic
44	14.01	3,5-Dicaffeoyl quinic acid	C25H24O12	[M-H]^-^	515.1201	515.1195	1.2	355.0894; 191.0565; 179.0359; 135.0444	Cynanchum otophyllum
45	14.61	2-O-Caffeoyl arbutin	C21H22O10	[M-H]^-^	433.1128	433.1140	-2.8	433.1067; 271.0576; 177.0185; 151.0028;119.0500	Fraxinus bungeana
46	14.84	4,5-Dicaffeoyl quinic acid	C25H24O12	[M-H]^-^	515.1205	515.1195	1.9	515.1185; 353.0891; 191.0581; 179.0346; 173.0454; 135.0462	Cynanchum otophyllum
47	14.93	Salvianolic acid E	C36H30O116	[M-H]^-^	717.1466	717.1461	0.7	717.1361; 519.0920; 339.0496; 321.0396; 295.0579; 279.0427; 197.0415	Radix Salviae miltiorrhizae
48	15.02	Paeoniflorin isomer	C23H28O11	[M+FA-H]^-^	525.1604	525.1614	-1.8	525.1603; 479.1536; 121.0291	Radix Paeoniae Alba
				[M+Na]+	503.1529	503.1524	1.0	503.1536; 381.1242; 341.1002; 219.0640	
49	15.36	Rosmarinic acid	C18H16O8	[M-H]^-^	359.0774	359.0772	0.4	359.0757; 197.0443; 179.0371; 161.0230; 133.0293; 72.9927	Radix Salviae miltiorrhizae
50	15.88	Salvianolic acid A isomer	C26H22O10	[M-H]^-^	493.1146	493.1140	1.2	493.1211; 313.0726; 295.0628; 253.0502; 185.0271; 159.0460; 109.0288	Radix Salviae miltiorrhizae
51	16.17	Licuraside	C26H30O13	[M-H]^-^	549.1594	549.1614	-3.6	549.1611; 417.1174; 255.0661; 135.0072; 91.0184	Radix liquiritiae
52	16.28	Buddleoside	C28H32O14	[M-H]^-^	591.1721	591.1719	0.3	591.1748; 549.1602; 459.1312; 255.0652; 135.0089	Radix isatidis
53	16.45	Ononin	C22H22O9	[M+FA-H]^-^	475.1252	475.1246	1.3	267.0649; 2224.0498	Curcuma aromatic
				[M+Na]+	431.1337	431.1337	0.1	269.0809; 254.0543; 213.0892	
54	16.85	Liquiritin isomer	C21H22O9	[M-H]^-^	417.1188	417.1191	-0.7	417.1189; 255.0643; 148.0160; 135.0091; 119.0491; 92.0246	Radix liquiritiae
				[M+H]+	419.1336	419.1337	-0.1	257.0812; 239.0707; 147.0437; 137.0221	
55	17.19	Salvianolic acid B	C36H30O16	[M-H]^-^	717.1482	717.1461	2.9	739.1302; 559.0863; 515.0974; 335.0553; 291.0662; 159.0476	Radix Salviae miltiorrhizae
				[M+Na]+	741.1413	741.1426	-1.8	741.1432; 561.1045; 543.0893; 517.1098; 381.0592; 363.0459; 337.0685; 221.0402	
56	17.84	Licorice-giycoside B	C35H36O15	[M-H]^-^	695.1981	695.1981	-0.1	695.1943; 549.1603; 399.1013; 255.0625	Radix liquiritiae
57	17.89	Licorice-giycoside A	C36H38O16	[M-H]^-^	725.2089	725.2087	0.3	725.2075; 549.1691; 531.1523; 399.1117; 255.0649; 193.0486; 119.0499; 72.9902	Radix liquiritiae
58	18.14	Methylnissolin-3-O-glucoside	C23H26O10	[M+FA-H]^-^	507.1500	507.1508	-1.6	/	Curcuma aromatic
59	18.15	Liquiritigenin	C15H12O4	[M-H]^-^	255.0668	255.0663	2.0	255.2316; 219.8452; 201.8352; 166.8654; 119.0503; 91.0173	Radix liquiritiae
				[M+H]+	257.0810	257.0808	0.6	257.0820; 242.0593; 153.0696; 147.0458; 137.0233; 119.0495; 81.-334	
60	18.46	9,10-DiMP-3-O-acetyl-Glc	C25H28O11	[M+Na]+	521.1077	521.1054	-0.5	521.1135; 493.1161; 341.0643; 323.0554; 295.0588; 277.0514; 249.0541; 181.0483; 163.0387; 139.0385; 111.0480	Curcuma aromatic
61	18.58	Salvianolic acid L	C36H30O16	[M-H]^-^	717.1478	717.1461	2.4	717.1490; 519.0934; 339.0504; 321.0401; 295.0603; 279.0275; 185.0240	Radix Salviae miltiorrhizae
62	18.9	Pectolinarin	C29H34O15	[M+H]+	623.1970	623.1970	-0.1	623.2009; 477.1407; 315.0876; 300.0637	Curcuma aromatic
63	18.94	Salvianolic acid Y	C36H30O16	[M-H]^-^	717.1476	717.1461	2.2	717.1451; 673.1693; 519.0950; 339.0534; 321.0403; 295.0644; 249.0569; 185.0238; 109.0279	Radix Salviae miltiorrhizae
64	18.98	Salvianolic acid C	C26H20O10	[M-H]^-^	491.0994	491.0984	2.1	491.1013; 311.0580; 293.0470; 267.0648; 250.0631; 197.0463; 135.0456	Radix Salviae miltiorrhizae
65	19.05	Salvianolic acid A or isomer	C26H22O10	[M-H]^-^	493.1128	493.1140	-2.5	493.1165; 313.0737; 295.0611; 185.0238; 159.0442; 109.0287	Radix Salviae miltiorrhizae
				[M+Na]+	517.1101	517.1105	-0.8	517.1129; 319.0485; 297.0763; 251.0721; 223.0743; 221.0433; 205.0626; 152.0622; 131.0527	
66	19.24	Calycosin	C16H12O5	[M-H]^-^	283.0615	283.0612	1.1	283.0622; 268.0406; 239.0352; 211.0388; 197.9039; 148.02229	Curcuma aromatic
				[M+H]+	285.0763	285.0758	1.9	285.0742; 270.0505; 253.0485; 213.0542; 197.0594; 137.0230; 89.0370	
67	19.37	Quercetin	C15H10O7	[M-H]^-^	301.0337	301.0354	-5.6	301.0323; 151.0022	Fraxinus bungeana, Hawthorn, Medicated Leaven
68	22.20	22-hydroxyl-licorice-saponin G2	C42H62O18	[M-H]^-^	853.3820	853.3805	1.8	853.3850; 351.0586	Radix liquiritiae
69	22.90	Licoricesaponin A3	C48H72O21	[M-H]^-^	983.4447	983.4449	-4.7	983.4515; 821.4051; 351.0644; 175.0356	Radix liquiritiae
70	23.54	Glyyunnanprosapogenin D or isomer	C42H62O17	[M-H]^-^	837.3920	837.3914	0.7	837.3943; 351.0601	Radix liquiritiae
71	24.20	Glyyunnanprosapogenin D or isomer	C42H62O17	[M-H]^-^	837.3874	837.3914	-4.8	837.3881; 351.0565	Radix liquiritiae
72	24.46	Glyyunnanprosapogenin D or isomer	C42H62O17	[M-H]^-^	837.3934	837.3914	2.4	837.3960; 351.0556	Radix liquiritiae
73	24.53	16-Oxoalisol A	C30H48O6	[M+H]+	505.3529	505.3524	1.1	505.3522; 415.2821; 353.2462; 191.1445; 107.0845	Rhizoma alismatis
74	24.7	Glycyrrhizic Acid	C42H62O16	[M-H]^-^	821.3988	821.3965	2.8	821.3988; 351.0554	Radix liquiritiae
				[M+Na]+	845.3940	845.3930	1.2	845.3945; 669.3590; 493.3277; 375.0511	
75	24.94	alisol C 23-acetate	C32H48O6	[M+H]+	529.3525	529.3524	0.3	/	Rhizoma alismatis
76	25.03	Licorice saponin B2	C42H64O15	[M-H]^-^	807.4134	807.4172	-4.8	807.4152; 351.0538	Radix liquiritiae
77	25.18	Uraisaponin B	C42H62O16	[M-H]^-^	821.3988	821.3965	2.8	821.4015; 351.0589	Radix liquiritiae
78	25.16	Glycyrrhetinic acid Monoglucuronide	C36H54O10	[M+H]+	647.3785	647.3790	-0.7	647.3812; 453.3368; 435.3196; 407.3384; 253.1876; 217.1558; 177.1634; 149.1341	Radix liquiritiae
79	25.38	Glycyrrhizic Acid isomer	C42H62O16	[M-H]^-^	821.3981	821.3965	1.9	821.4016; 351.0611	Radix liquiritiae
80	25.63	alisol C 23-acetate	C32H48O6	[M+H]+	529.3525	529.3524	0.3	529.3536; 511.3355; 469.3326; 451.3232; 415.2877; 217.1586	Rhizoma alismatis
81	26.31	Demethoxycurcumin	C20H18O5	[M-H]^-^	337.1060	337.1081	-6.4	/	Curcuma aromatic
82	26.48	curcumin	C21H20O6	[M-H]^-^	367.1172	367.1187	-4.1	367.1182; 309.-398; 241.0083; 203.-723; 173.0237; 59.0105	Curcuma aromatic
83	26.80	alisol C	C30H46O5	[M+H]+	487.3418	487.3418	0.1	487.3419; 451.3200; 433.3082; 397.2727; 353.2452; 175.1108; 147.1156	Rhizoma alismatis
84	27.36	Astragaloside I	C45H72O16	[M-H]^-^	913.4825	913.4802	2.5	913.4793; 867.4743	Curcuma aromatic
85	27.63	Licoisoflavone A	C20H18O6	[M-H]^-^	353.1021	353.1031	-2.7	353.0998; 125.0346	Radix liquiritiae
86	27.94	Dimethyldibenzylidene Sorbitol	C24H30O6	[M+H]+	415.2120	415.2115	1.2	119.0853; 115.0516; 91.0545	Rhizoma alismatis
87	28.05	tanshinoneII A	C19H20O3	[M+H]+	297.1488	297.1485	0.9	297.1413; 253.1594; 222.0666; 1666.0784; 128.0643; 73.0466	Radix Salviae miltiorrhizae
88	28.77	dihydrotanshinone I	C18H14O3	[M+H]+	279.1020	279.1020	1.5	279.0990; 261.0918; 233.0961; 190.0759; 169.0641; 141.0687; 115.0537	Radix Salviae miltiorrhizae
89	30.50	alisol B	C30H48O4	[M+H]+	473.3628	473.3625	0.6	/	Rhizoma alismatis
90	31.54	cryptotanshinone	C19H20O3	[M+H]+	297.1490	297.1485	1.6	297.1471; 268.1102; 236.1164; 209.0977; 165.0714; 155.0923	Radix Salviae miltiorrhizae

### The Effect of Qianggan Extract on Hyperglycemia in Rats

**Figure 2:**
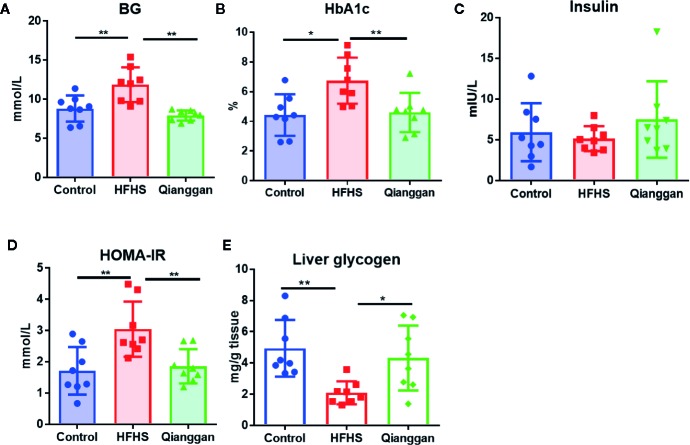
Effects of Qiangggan extract on hyperglycemia. Hyperglycemia was induced by HFHS feeding, Qianggan extract were treated for 4 weeks. **(A)** Fasting blood glucose **(B)** HbA1c, **(C)** Insulin, **(D)** HOMA-IR, **(E)** Liver glycogen. Data were presented as mean ± SD, ^*^ *p* < 0.05, ^**^ *p* < 0.001.

Rats feeding HFHS diet showed hyperglycemia, as the blood glucose was significantly increased compared with chow diet control rats (**Figure 2A**). Four-week Qianggan extract treatment restored the blood glucose increase to normal level (**Figure 2A**). Similar trend was also observed in HbAlc levels (**Figure 2B**). Although the insulin level has no statistical difference among groups (**Figure 2C**), HOMA-IR was significantly increased in HFHS rats (**Figure 2D**), and Qianggan extract treatment markedly reduced HOMA-IR value. Glucose can be stored in the form of glycogen in liver, and liver glycogen is critical in maintaining glucose homeostasis (von Wilamowitz-Moellendorff et al., 2013). We found obviously decreased liver glycogen in HFHS rats, and Qianggan extract treatment significantly increased liver glycogen content (**Figure 2E**). Qianggan extract treatment also partially restored the increased serum ALT and AST levels in HFHS rats, however, the body weight, liver weight, and serum lipids did not show statistical difference among groups (**Table 2**).

**Table 2:** Phenotypic parameters of the rats.

Parameters	Control	HFHS	Qianggan
**Body weight (g)**	385.90 ± 27.29	403.50 ± 29.24	381.90 ± 26.48
**Liver weight (g)**	9.17 ± 0.92	9.49 ± 0.71	8.97 ± 1.13
**Serum ALT**	34.93 ± 5.32	56.01 ± 31.07^*^	30.61 ± 7.46^‡^
**Serum AST**	150.50 ± 19.39	191.00 ± 45.92^*^	118.60 ± 19.21^‡^
**Serum TG**	0.88 ± 0.31	0.61 ± 0.26^**^	0.52 ± 0.19
**Serum TC**	1.42 ± 0.17	1.29 ± 0.08	1.50 ± 0.15
**Serum HDL-c**	0.57 ± 0.06	0.51 ± 0.03^*^	0.52 ± 0.09
**Serum LDL-c**	0.13 ± 0.17	0.02 ± 0.01	0.17 ± 0.05

* P < 0.05, ^**^P < 0.05, HFHS vs control; ^‡^P < 0.05 Qianggan vs HFHS.

### Metabolite Profile and Differential Metabolites Identification

**Figure 3:**
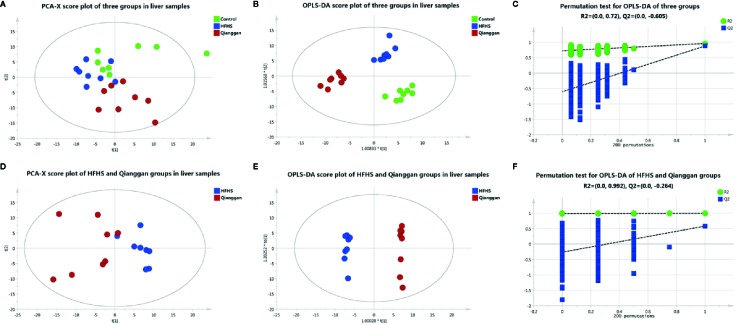
Multivariate analysis based on metabolomics of liver samples**. (A)** PCA score plot among control, HFHS diet, and Qiangggan groups. R2X=0.648, Q2 = 0.277; **(B)** OPLS-DA score plot among three groups. R2X=0.512, R2Y= 0.913, Q2 = 0.277; **(C)** 200 permutation tests validation of OPLS-DA among three groups. R2 = 0.72, Q2=-0.605; **(D)** PCA score plot between HFHS diet and Qiangggan groups. R2X=0.622, Q2 = 0.149; **(E)** OPLS-DA score plot between HFHS diet and Qiangggan groups. R2X=0.584, R2Y= 0.998, Q2 = 0.582; **(F)** 200 permutation tests validation of OPLS-DA between HFHS diet and Qiangggan groups. R2 = 0.992, Q2=-0.264.

To unravel the mechanisms under the efficacy of Qianggan extract, metabolomics were conducted to obtain metabolite profiles and identify differential metabolites in liver tissue and fecal samples. The GC-MS chromatograms of liver and fecal samples were presented in **Supplemental Figure 1**. PCA and OPLS-DA models were established to visualize clusters and different metabolic patterns among groups. For liver tissues, PCA model did not clearly separate control, HFHS and Qianggan groups (**Figure 3A**). However, OPLS-DA model revealed good separation among three groups (**Figure 3B**). Parameters of R2X=0.512, R2Y= 0.913, and Q2 = 0.277, indicating the good quality and accurate prediction of the model. Two hundred permutation tests were further performed, with R2 = 0.72 and Q2=-0.605, suggesting the reliability of the OPLS-DA model (**Figure 3C**). To identify differential metabolites between HFHS diet and Qianggan treated groups, PCA and OPLS-DA models were built. PCA did not clearly discriminate the two groups, but a good separation was observed by OPLS-DA plots (**Figures 3D, E**), implicating Qianggan extract improved metabolite perturbations induced by HFHS diet. Permutation test implicated the validity of OPLS-DA model with R2 = 0.992 and Q2=-0.264 (**Figure 3F**). Moreover, metabolites with VIP value > 1 were obtained. Coupled with univariate statistical analysis (*p* < 0.05), 18 metabolites (e.g. glucose-6-phosphate, fructose-6-phosphate and ribose-5-phosphate) were identified to be significantly different between HFHS diet and Qianggan treated groups (**Table 3**).

**Table 3:** Significantly different metabolites in liver tissues.

rt/min	m/z	metabolites	VIP	P value	Log_2_(fold change) Qianggan *vs* HFHS
17.26	174	gamma-aminobutyric acid	1.313	0.038	0.559
22.22	103	fructose	1.774	0.038	-0.969
22.38	319	mannose	1.723	0.026	-0.735
22.92	205	mannitol	1.579	0.029	-0.651
28.42	361	lactose	1.495	0.038	-0.745
20.89	357	glycerol-3-phosphate	1.554	0.026	-0.290
12.5	174	glycine	1.854	0.029	0.413
6.96	219	lactic acid	1.733	0.030	0.572
26.37	387	glucose-6-phosphate	1.603	0.026	-1.447
26.29	315	fructose-6-phosphate	1.478	0.033	-0.930
7.32	177	glycolic acid	1.557	0.019	0.685
22.92	333	glucuronic acid	1.676	0.026	-0.662
27.69	387	sedoheptulose-7-phosphate	1.646	0.019	-0.974
24.65	315	ribose-5-phosphate	1.383	0.019	-0.493
8.43	131	2-hydroxybutyric acid	1.707	0.026	0.717
24.61	441	uric acid	1.614	0.037	1.727
28.6	361	maltose	1.673	0.026	-0.753
23.08	333	galacturonic acid	1.581	0.050	-0.486

**Figure 4:**
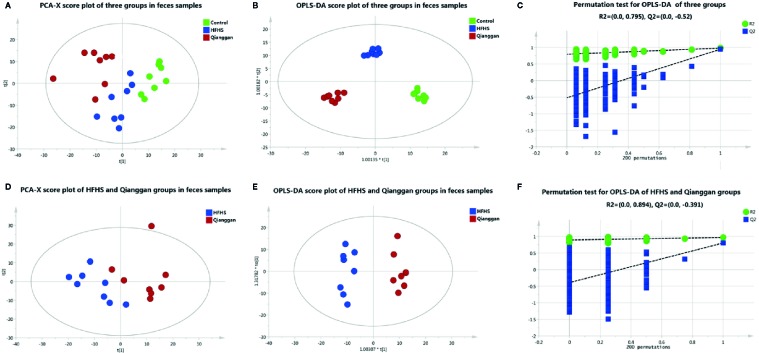
Multivariate analysis based on metabolomics of fecal samples. **(A)** PCA score plot among control, HFHS diet and Qiangggan groups. R2X=0.612, Q2 = 0.327; **(B)** OPLS-DA score plot among three groups. R2X=0.634, R2Y= 0.976, Q2 = 0.858; **(C)** 200 permutation tests validation of OPLS-DA among three groups. R2 = 0.795, Q2=-0.52; **(D)** PCA score plot between HFHS diet and Qiangggan groups. R2X=0.581, Q2 = 0.204, **(E)** OPLS-DA score plot between HFHS diet and Qiangggan groups. R2X=0.527, R2Y= 0.968, Q2 = 0. 802; **(F)** 200 permutation tests validation of OPLS-DA between HFHS diet and Qiangggan group. R2 = 0.894, Q2=-0.391.

Same analyses in fecal samples were performed (**Figure 4**). OPLS-DA plots demonstrated clear separations among three groups (control, HFHS diet and Qianggan treated groups) and in pairwise groups (HFHS diet *vs* Qianggan intervened groups). Permutation test showed good prediction of the model. By the cutoff of VIP > 1 and *p* < 0.05, we obtained 30 differential metabolites (e.g. maltose, glycolic acid, and 4-hydroxyproline), suggesting Qianggan extract ameliorated HFHS diet induced metabolite disturbance in feces. Detailed metabolite information was listed in **Table 4**.

**Table 4:** Significantly different metabolites in fecal samples.

rt/min	m/z	metabolites	VIP	P value	Log_2_(fold change) Qianggan vs HFHS
9.61	187	heptanoic acid	1.565	0.007	-1.943
28.58	361	maltose	1.383	0.017	-1.576
17.97	267	3-hydroxybenzoic acid	1.340	0.018	-1.491
19.9	260	N-methylglutamic acid	1.372	0.009	-1.477
19.29	103	lyxose	1.439	0.015	-1.382
19.54	103	arabinose	1.296	0.043	-1.378
16.56	202	p-hydroxybenzaldehyde	1.487	0.010	-1.376
25.96	144	spermidine	1.505	0.007	-1.284
23	333	glucuronic acid	1.516	0.007	-1.187
14.98	104	hydrocinnamic acid	1.383	0.027	-1.059
20.92	292	lyxonic acid	1.337	0.043	-0.961
16.06	218	aminomalonic acid	1.391	0.019	-0.873
17.22	230	4-hydroxyproline	1.332	0.024	-0.866
20.3	117	rhamnose	1.236	0.015	-0.807
11.7	174	ethanolamine	1.241	0.033	0.481
24.88	352	guanine	1.267	0.026	0.555
24.52	217	myo-inositol	1.475	0.006	0.698
25.08	327	heptadecanoic acid	1.371	0.026	0.725
21.61	273	citric acid	1.342	0.020	0.750
7.67	205	glycolic acid	1.197	0.041	0.840
9.08	219	3-hydroxypropanoic acid	1.518	0.009	0.860
15.83	174	3-aminoisobutanoic acid	1.121	0.033	0.962
17.29	304	gamma-aminobutyric acid	1.597	0.006	1.067
8.82	219	oxalic acid	1.663	0.004	1.071
20.77	142	ornithine	1.386	0.026	1.186
23.26	299	pentadecanoic acid	1.458	0.015	1.267
19.64	202	asparagine	1.549	0.006	1.417
31.5	329	cholesterol	0.985	0.026	1.771
9.27	165	p-cresol	1.502	0.007	1.827
30.76	370	coprostanol	1.169	0.009	1.845

**Figure 5:**
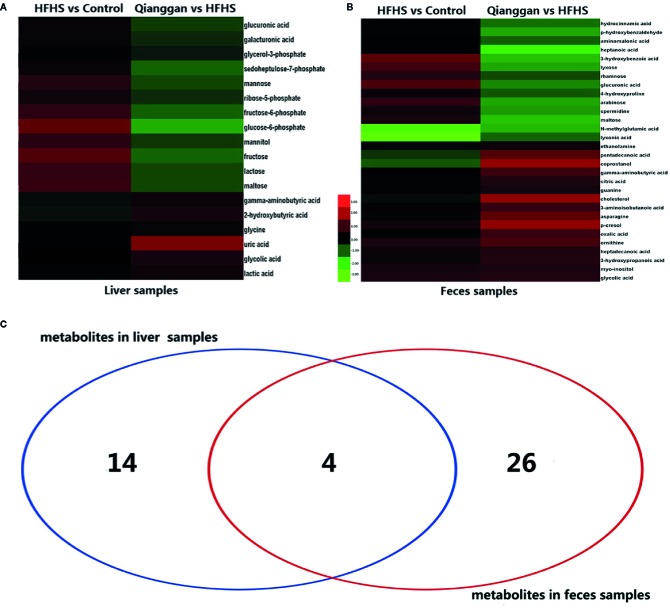
Significantly different metabolites among groups. **(A)** Hierarchical cluster analysis between pairwise groups (HFHS *vs* control and Qianggan *vs* HFHS) for identified metabolites from liver samples. **(B)** Hierarchical cluster analysis of identified metabolites between pairwise groups (HFHS *vs* control and Qianggan *vs* HFHS) in fecal samples. **(C)** Venn diagram to reveal overlapped and gross metabolites obtained from liver and fecal samples. Red color represents up-regulation and green represents down-regulation.

To better visualize the patterns of differential metabolites, hierarchical clusters were performed. As shown in **Figures 5A, B**, distinct discrimination can be observed in pairwise groups in both liver and fecal samples. Of interest, most metabolites are in opposite pattern between HFHS *vs* Control and Qiangggan *vs* HFHS. For instance, glucose-6-phosphate and fructose-6-phosphate levels were higher in HFHS diet group compared to control group, but significantly decreased in Qiangggan intervened group. The data implicated that Qianggan extract markedly restored HFHS diet induced metabolites disturbance, and the affected metabolites might be potential targets of the compound. By Venn diagram (**Figure 5C**), we observed four overlapped metabolites between liver and fecal samples. In all, we obtained 44 potential metabolites used for further analysis.

### MSEA and Metabolic Pathway Analysis

**Figure 6:**
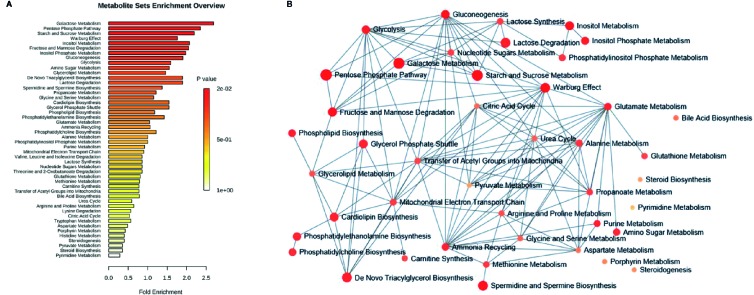
MSEA and pathway enrichment overview. **(A)** MSEA overview obtained through MetaboAnalyst 4.0 by plotting -log of p-values from pathway enrichment analysis on the y-axis, and pathway impact values from pathway topology analysis on the x-axis. **(B)** Pathway interaction network graph obtained by MetaboAnalyst 4.0 enrichment analysis. Nodes represent different enriched pathways and edges represent correlations.

**Figure 7:**
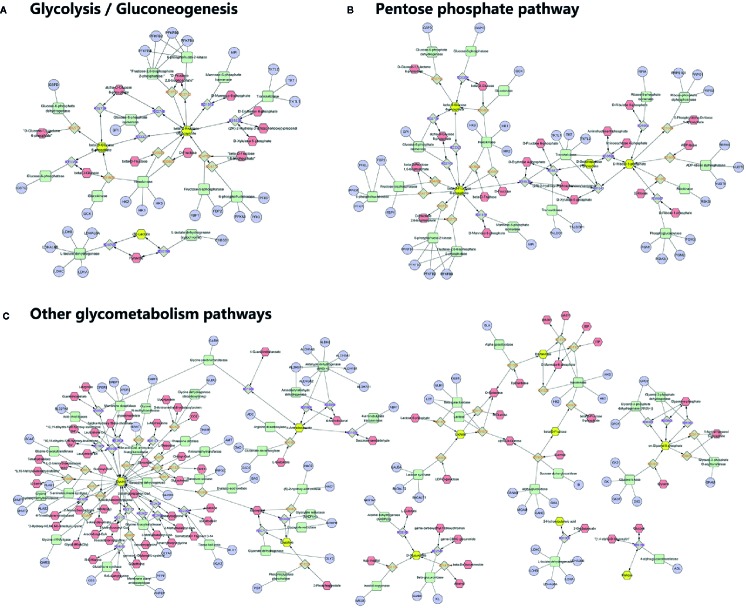
Compound-reaction-enzyme-gene network analysis for enriched pathways. **(A)** Network in glycolysis/gluconeogenesis, **(B)** Network in Pentose phosphate pathway, **(C)** Network in other related pathways. Yellow hexagons represent identified differential metabolites in relevant metabolic pathways. Red hexagons represent intermediates might related with the identified metabolites. Green squares represent enzymes which might regulate the identified metabolites. Blue circles represent genes encoding those enzymes. Grey diamonds represent reactions catalyzed by those enzymes.

To understand the biological meaning and relevant metabolic pathways of the identified 44 metabolites, comprehensive MSEA and pathway enrichment analysis were performed. As shown in **Figure 6**, these metabolites were enriched in 43 metabolic pathways, and the top 10 were all glycometabolism related pathways (e.g. glycolysis/gluconeogenesis, pentose phosphate pathway, fructose and mannose metabolism, *etc*), and the alteration of these pathways might account for the efficacy of Qianggan extract on hyperglycemia. Of note, these metabolic pathways interconnected with each other and formed a complex network. Furthermore, to understand the complicated correlations among genes, enzymes, and metabolites in enriched pathways, we constructed the compound-reaction-enzyme-gene network (**Figure 7**). For instance, the metabolite glucose-6-phosphate was disturbed by HFHS diet and improved by Qiangggan extract, and predictably, related genes (e.g. *Gck, Hk1, Hk2*, *etc*) and enzymes (e.g. glucokinase, hexokinase, *etc*) in glycolysis/gluconeogenesis pathway were involved in the regulation process.

## Discussion

In the present study, we illustrated the effect of Qiangggan extract on diet induced hyperglycemia, and through the analysis of metabolomics, we identified glycometabolism related pathways were involved in the metabolic disturbance and under the benefit effects of Qianggan extract.

Metabolomics has been extensively employed in detecting metabolites profiles to explore the pathophysiology of diseases, predict potential biomarkers, and identify drug targets (Sun et al., 2014). The balance of glucose metabolism was impaired in patients with liver injury (Guo et al., 2015) and steatosis (Hu et al., 2018).

Glycolysis and gluconeogenesis are critical pathways in keeping glucose balance (Petersen et al., 2017). Glycolysis is a glucose utilization process, which converts glucose into pyruvate or lactate. Gluconeogenesis is opposite to that of glycolysis, which synthesizes glucose from other metabolites like pyruvate, lactate, and glucogenic amino acids (Tang et al., 2018). Glycolysis and gluconeogenesis possess several reversible enzyme-catalyzed reactions and share a series of common intermediates such as glucose-6-phosphate, fructose-6-phosphate, fructose-1, 6-bisphosphate, lactate, *etc* (Sharabi et al., 2015). The net flux toward glycolysis or gluconeogenesis may be regulated by the key enzymes or their related metabolites which could be influenced by multi-factors such as nutrients and drugs. Using metabolomics approach, Wan et al. reported several intermediates including fructose 6-phosphate and 6-phospho-gluconate were elevated in high fat diet fed rats liver, and the alteration was reversed by vine tea, which implicated the efficacy partially by altering glycolysis or gluconeogenesis (Wan et al., 2017). It is also reported that HFHS diet could induce accelerated gluconeogenesis to yield glucose (Commerford et al., 2001). Our data were in accordance with previous studies to some extent. We noticed that glycolysis or gluconeogenesis intermediates glucose-6-phosphate and fructose-6-phosphate were raised after HFHS diet feeding. Qianggan extract administration restored the increase of glucose-6-phosphate and fructose-6-phosphate and raised lactic acid, implicating that Qiangggan extract improved glucose metabolism disorders partially by accelerating glycolysis or suppressing gluconeogenesis. Similar results were also exhibited in another insulin resistance rat model, which reported that coreopsis tinctoria flowering tops (traditionally employed to improve hyperglycemia) could reduce the increase of fructose 6-phosphate and 6-phosphogluconate induced by high fat diet (Jiang et al., 2015).

Pentose phosphate pathway branches from glycolysis *via* glucose-6-phosphate at the first committed step (Cho et al., 2018). Dong et al. employed metabolomics to explore biomarkers of different stage of nonalcoholic fatty liver disease (NAFLD) and demonstrated that pentose phosphate pathway was involved in the progress of NAFLD (Dong et al., 2017). Another study reported that pentose phosphate pathway was related to diabetes retinopathy and relevant metabolites were increased (Chen et al., 2016). In the present study, hyperglycemia status showed elevated metabolites that related to pentose phosphate pathway, such as glucose-6-phosphate, ribose-5-phosphate, and sedoheptulose-7-phosphate, which were attenuated by Qianggan extract. Our data were partly in line with previous studies (Hong et al., 2017), suggested the alteration of pentose phosphate pathway more or less account for the efficacy of Qiangggan extract. Besides, glycogenesis (glycogen synthesis) is reliant on glycolysis and starts with glucose-6-phosphate, is the process of glucose storage and vital in the maintenance of glucose concentration (Han et al., 2016). It was reported that glycogen content was decreased in high fat diet induced obese rats, and improved by octreotide which might serve as a novel treatment of obesity (Wang et al., 2017). Our data showed that the level of glycogen was significantly lowered in hyperglycemia and improved after Qiangggan extract intervention, which were consistent with the previous studies.

In addition, fructose and mannose metabolism also disturbed under metabolic dysfunctions. Zhang et al. found metabolites fructose and mannose were markedly elevated, which were deemed to be potential biomarkers of type 2 diabetes in patients (Zhang et al., 2016a). Boztepe et al performed microarray analysis to explore molecular responses to high glucose, and identified fructose and mannose metabolism was altered (Boztepe and Gulec, 2018). The data suggested that fructose and mannose metabolism play a role in metabolic diseases. Consistently, we also observed increased metabolites of fructose, mannitol, and mannose in hyperglycemia group compared to control group, whereas, Qiangggan extract significantly lowered the expression of these metabolites, suggesting potential targets of Qianggan extract.

A compound-reaction-enzyme-gene network was visualized to help in understanding the complex relations among metabolites, proteins, or genes in relevant metabolic pathways. For instance, we noticed that hexokinase (encoded by genes *Hk1, Hk2* and *Hk3*) and glucokinase (encoded by *Gck*) might regulate glucose-6-phosphate. Besides hexokinase, many other enzymes such as fructose-bisphosphatase (encoded by *Fbp1* and *Fbp2*) and mannose-6-phosphate isomerase (encode by *Mpi*) were related to the regulation of fructose-5-phosphate. Several enzymes such as ribokinase (encoded by *Rbks*) and ribose-5-phosphate isomerase (encoded by *Rpia*) may play a role in modulating the level of ribose-5-phosphate. Our findings were corroborated by abundant previous studies. For example, the activators of the enzyme glucokinase which converts glucose to glucose-6-phosphate in glycolysis, could ameliorate hyperglycemia and have been used as novel glucose-lowering drugs in diabetic models (Erion et al., 2014; Rubtsov et al., 2015). The enzyme ribose-5-phosphate isomerase was correlated with live cancer and has been identified as potential target of therapy (Ciou et al., 2015). Further investigations on identified metabolites and their related enzymes may ascertain Qiangggan extract targets and obtain novel therapies to treat high glucose related diseases.

Notably, the dosage of Qianggan extract for alleviating hyperglycemia was two times of the dosage used for improving fatty liver disease in rats, but the proper dosage for human needs to be optimized in the clinical settings. Our data were based on GC-MS metabolomics. We identified potential metabolites, relevant pathways, and key enzymes, however, we did not detect the expressions of correlated genes or enzymes in specific metabolic pathways. Comprehensive investigation of transcriptomics or proteomics and drug-metabolites interactions should be performed to mutually validate our finding from metabolomics (Ge, 2019; Zhou et al., 2019). In addition, our findings were obtained from animal models, and massive experiments and clinical investigations should be employed to further verify the data and for later clinical translation.

## Conclusion

Qiangggan extract restored diet-induced glucose metabolism perturbations. The efficacy might partially due to the regulation of relevant glycometabolism pathways such as glycolysis or gluconeogenesis, pentose phosphate pathway, glycogenesis, fructose, and mannose metabolism. Our findings may infer the potential mechanisms of Qianggan extract on hyperglycemia.

## Data Availability Statement

The raw data supporting the conclusions of this article will be made available by the authors, without undue reservation, to any qualified researcher.

## Ethics Statement

All animal procedures were approved by the Animal Experiment Ethics Committee of Shanghai University of Traditional Chinese Medicine, and the approval number is PZSHUTCM191227006.

## Author Contributions

GJ and LZ designed the study. ML, WZ, and LZ performed the experiment. GG performed the chemical profiling. MZ analyzed the data. MZ, LZ, and GJ wrote the manuscript.

## Funding

This work was supported by National Natural Science Foundation of China (No. 81620108030, 81774084). The funders had no role in study design, data collection and analysis, decision to publish, or preparation of the manuscript.

## Conflict of Interest

The authors declare that the research was conducted in the absence of any commercial or financial relationships that could be construed as a potential conflict of interest.
