# Supplementary material for: Metabolomic Analysis Identifies Glycometabolism Pathways as Potential Targets of Qianggan Extract in Hyperglycemia Rats
Source: Front Pharmacol. 2020 May 12;11:671. doi: 10.3389/fphar.2020.00671 (PMC7235344; doi:10.3389/fphar.2020.00671)
Supplement: Supplementary file 1 [file DataSheet_1.docx]

Supplementary Material

## Supplementary Figures


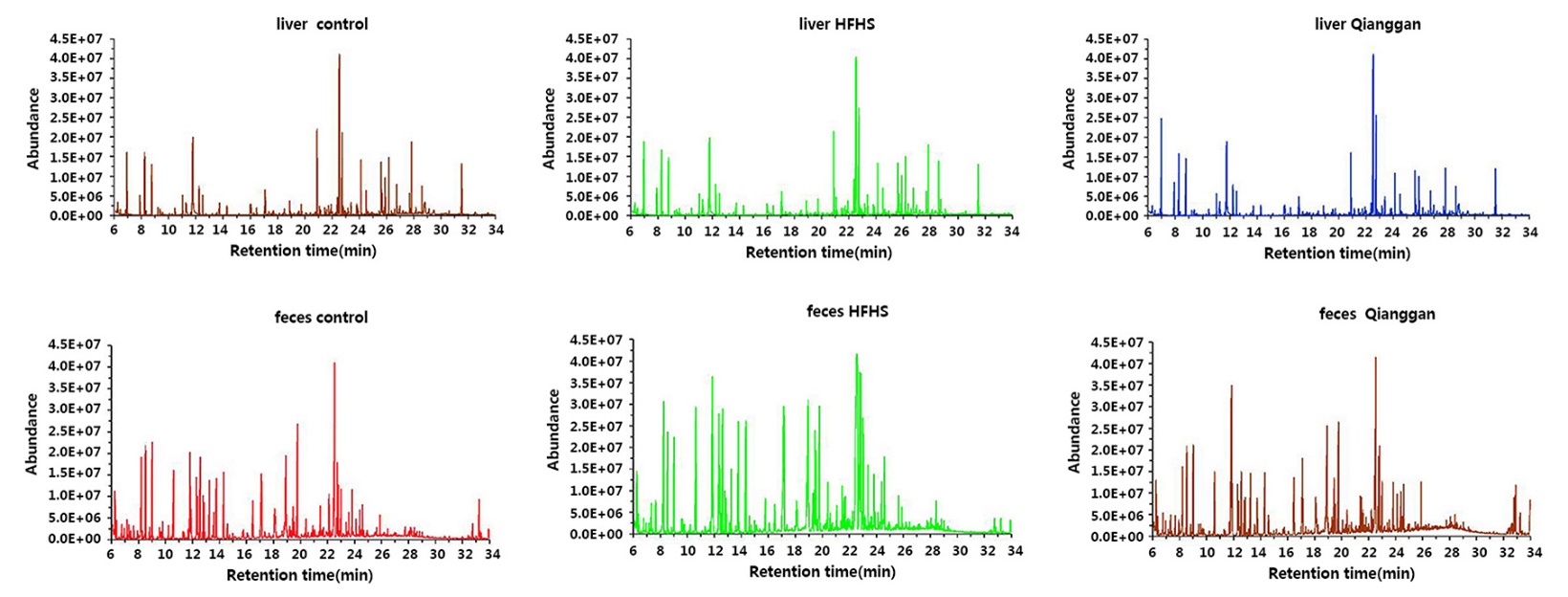


**Supplementary Figure 1.** The GC-MS chromatograms of liver and fecal samples.
